# High Rates of Ofloxacin Resistance in *Mycobacterium tuberculosis* among Both New and Previously Treated Patients in Tamil Nadu, South India

**DOI:** 10.1371/journal.pone.0117421

**Published:** 2015-03-04

**Authors:** N. Selvakumar, Vanaja Kumar, S. Balaji, S. Prabuseenivasan, R. Radhakrishnan, Gomathi Sekar, V. Chandrasekaran, T. Kannan, Aleyamma Thomas, S. Arunagiri, Puneet Dewan, Soumya Swaminathan

**Affiliations:** 1 National Institute for Research in Tuberculosis (Indian Council of Medical Research), Chennai, India; 2 State TB Cell, Government of Tamil Nadu, Chennai, India; 3 WHO India, New Delhi, India; University of Delhi, INDIA

## Abstract

Periodic drug resistance surveillance provides useful information on trends of drug resistance and effectiveness of tuberculosis (TB) control measures. The present study determines the prevalence of drug resistance among new sputum smear positive (NSP) and previously treated (PT) pulmonary TB patients, diagnosed at public sector designated microscopy centers (DMCs) in the state of Tamil Nadu, India. In this single-stage cluster-sampling prevalence survey, 70 of 700 DMCs were randomly selected using a probability-proportional to size method. A cluster size of 24 for NSP and a varying size of 0 to 99 for PT cases were fixed for each selected DMC. Culture and drug susceptibility testing was done on Lowenstein-Jensen medium using the economic variant of proportion sensitivity test for isoniazid (INH), rifampicin (RMP), ofloxacin (OFX) and kanamycin (KAN). Human Immunodeficiency Virus (HIV) status was collected from patient records. From June 2011 to August 2012, 1524 NSP and 901 PT patients were enrolled. Any RMP resistance and any INH resistance were observed in 2.6% and 15.1%, and in 10.4% and 30% respectively in NSP and PT cases. Among PT patients, multi drug resistant TB (MDR-TB) was highest in the treatment failure (35%) group, followed by relapse (13%) and treatment after default (10%) groups. Extensively drug resistant TB (XDRTB) was seen in 4.3% of MDR-TB cases. Any OFX resistance was seen in 10.4% of NSP, 13.9% of PT and 29% of PT MDR-TB patients. The HIV status of the patient had no impact on drug resistance levels. RMP resistance was present in 2.6% of new and 15.1% of previously treated patients in Tamil Nadu. Rates of OFX resistance were high among NSP and PT patients, especially among those with MDR-TB, a matter of concern for development of new treatment regimens for TB.

## Introduction

Tuberculosis (TB) caused by *Mycobacterium tuberculosis* remains a major public health issue in most of developing countries, despite scaling up interventions to achieve global control [1]. The risk of progression of TB is enhanced by human immunodeficiency virus (HIV) infection and malnutrition, especially in Asia and Africa [2,3], besides social determinants like poor housing and poverty. In 2011, there were an estimated 8.7 million new cases of TB (13% co-infected with HIV) and 1.4 million TB deaths, including one million deaths among HIV-negative patients and 0.43 million among HIV-positive patients [4].

Emergence of multi drug resistant TB (MDR-TB: organism resistant to isoniazid [INH] and rifampicin [RMP]) is a major hurdle for TB control programs especially in developing countries like India. The global report on drug resistance surveillance by the World Health Organization (WHO) estimated that 3.6% of new smear positive TB (NSP) cases and 20% of previously treated cases (PT) have MDR-TB [5]. Of the total MDR-TB cases, 60% are in just three countries viz. India, China and Russia. Therefore, a regular national drug resistance surveillance programme is imperative to monitor the levels of drug resistance among NSP and PT cases, and to assess the performance of national TB control programmes [6].

While no national drug resistance survey has been undertaken in India, a few statewide surveys in Gujarat and Tamil Nadu during 2007 [7] and 1997 [8], respectively have been done. A drug resistance survey done in the state of Tamil Nadu a decade ago, reported any RMP resistance of 4.4% among NSP patients [8]. Approximately fifteen years after the Revised National TB Control program (RNTCP) was implemented, a study was initiated to determine the prevalence of MDR-TB and extensively drug resistant (XDR-TB) among new and previously treated pulmonary TB patients, diagnosed in public sector designated microscopy centres (DMCs). Further, we aimed to determine the prevalence of resistance to ofloxacin (OFX) and kanamycin (KAN), two drugs that form the backbone of second-line treatment, and which have not been investigated previously.

## Methodology

### a. Study design (cross-sectional cluster survey)

A population-based study was undertaken, which considered the annualized number of NSP and PT smear positive pulmonary TB cases diagnosed in each of the DMCs in the state of Tamil Nadu. To provide quality assured TB smear microscopy services with easy access for the entire population, a network of DMCs with competency in acid-fast sputum smear microscopy was established by the RNTCP. Each DMC caters to an approximate population of 1,00,000. A few private sector laboratories including non-governmental organisation (NGOs) also serve as DMCs in Tamil Nadu. The study period was from May 2011 to August 2012.

Recent WHO guidelines [9] for drug resistance surveillance (DRS) were followed with slight modifications. In brief, the prevalence of MDR-TB among NSP and PT cases, observed in Gujarat state, formed the basis for the present calculation [7]. The estimated sample size for NSP cases was 840. This was based on presumed 2% prevalence of MDR-TB among NSP cases, with a precision of 1% and an expected loss of 10%. The required sample size calculated was 1680 after applying the design effect of 2 for cluster sampling. For PT cases, the estimated sample size was 496. This was based on presumed 12% prevalence of MDR-TB among PT cases with a precision of 3% and an expected loss of 10%. The required sample size was calculated as 992 after applying the design effect of 2 for cluster sampling.

**Ethical statement**: The study was reviewed and approved by institutional ethics committee of National Institute for Research in Tuberculosis (NIRT), Chennai, India. Informed written patient consent was obtained from all patients enrolled in the study. The study was also reviewed and approved by scientific advisory committee of NIRT.

### b. Selection of patients

The state of Tamil Nadu has 700 DMCs. A random sample of 70 DMCs (10%) was selected from the list of 700 DMCs by probability-proportional to size (PPS) method [9]. A cluster size of 24 NSP was fixed for each of the selected DMC. Based on the annualized sample size of previous year for each DMC selected, 2 DMCs were assigned a cluster size of 48 for NSP cases; accordingly the total number of DMC included was 68 in numbers. While clusters of varying sizes (0–99) were fixed for PT patients for each of these DMCs selected [9]. The cluster size for PT patients was calculated based on the annualized sample size of previous year for each DMC selected. Consecutive eligible NSP and PT cases were enrolled. At DMC, sputum was stained by the hot Ziehl-Neelson method and examined as per the RNTCP procedures [10]. If the patient was sputum smear positive and belonged to the defined geographical area of the state, two additional spot sputum specimens were collected.

### c. Transport of sputum samples

Before the initiation of the study, an orientation programme was arranged at each district TB center (DTC) for health workers (Lab technician, Senior TB laboratory supervisor, DOTS plus supervisor, treatment supervisor, treatment organizer, Medical officer and District TB officer) involved in the study. A detailed presentation was made on study objectives, how to fill the clinical information form and informed patient consent form, and how to pack the sputum containers (triple packing system) and handover the packs to the courier agents for safe transportation. All the necessary materials for sputum collection and transportation were given, in a package, to the health workers of the participating DMCs during the orientation session.

Contrary to the conventional practice of collecting either 3 samples (spot-morning-spot strategy) or consecutive 2 or 3 morning samples, two spot sputum specimens were collected from eligible patients within 2–3 hours. In addition, cetyl pyridinium chloride (CPC) was not used to preserve sputum and ice packs were not used during transportation. Both specimens were carefully placed in a single pack following triple package system and transported at ambient temperature through courier.

### d. Culture and Drug susceptibility testing

Briefly, the specimens were processed by modified Petroff’s method and the deposits thus obtained were used for making smears for fluorescence microscopy and for culture on 2 Lowenstein Jensen (LJ) slopes. The slopes were incubated at 37°C for eight weeks. Culture growth was recorded every week. Identification of *M. tuberculosis* was done by growth on para-nitro benzoic acid (PNB) at 500 μg/ml and niacin test. Economic variant of proportion sensitivity test [11] was followed for susceptibility testing for two first line drugs, namely, INH (0.2 μg/ml) and RMP (40 μg/ml) and for two second line drugs, namely OFX (2 μg/ml) and KAN (30 μg/ml) [12]. As per the recent recommendation by WHO, susceptibility testing of *M. tuberculosis* was restricted to the two first line drugs (INH and RMP) and two second line drugs (OFX and KAN) [9].

### e. Analysis of data

Prevalence of drug resistance levels in NSP, PT patients (including its sub-groups; treatment failures, treatment after default and relapse), was determined and compared. The recovery of *M. tuberculosis* from the two samples was compared. Average time taken to transport sputum samples was calculated.

### f. Data Management and Statistical methods

Double-entry of data was done, verified and analyzed using SPSS version 14.0 (Statistical Package for the Social Sciences Inc, Chicago, IL, USA). Variables were expressed as percentages. Chi-square test was used to test the significant differences between groups and a P value less than 0.05 was considered statistically significant. Odds ratio and confidence intervals were calculated.

## Results

Of the total 2425 patients enrolled in the study, 1524 were NSP and 901 were PT cases. Men constituted 77% and 63% respectively among NSP and PT cases, and the median age was 45 years (range 15 to 65 years).

Of the 1524 NSP patients, samples from 17 were contaminated (1.1%), 7 grew non-tuberculous mycobacteria (0.5%), 78 were culture negative (5.1%) and the remaining 1422 yielded *M. tuberculosis* (93.3%). Similarly, of the 901 PT cases, samples from 7 were contaminated (0.8%), 16 had non-tuberculous mycobacteria (1.8%), 31 were culture negative (3.4%) and the remaining 847 (94%) grew *M. tuberculosis*. The recovery of *M. tuberculosis* from the study subjects was similar and high by using either of the two spot samples (recovery rate among 1^st^ spot specimen was 93.5% and second spot was 93.7%) or using both spot specimens (93.6%) [the McNamara’s test p value = 1.114]. The average time taken, after collection, for specimens to arrive in the laboratory was 1 day (range 1–15 days). Among culture positive isolates, drug susceptibility test results were available for 1220 NSP (85.8%) and 714 PT (84.3%) cases (Table 1).

**Table 1:** Mycobacterial culture results from patients enrolled in the survey.

	**New cases**		**Previously treated cases**		**TOTAL**	
	**(n = 1524)**		**(n = 901)**		**(n = 2425)**	
	**Numbers**	%	**Numbers**	%	**Numbers**	%
**Contamination (bacteria, fungi and others)**	17	1.1	7	0.8	24	1.0
**Non Tuberculous Mycobacteria**	7	0.5	16	1.8	23	0.9
***M. tuberculosis* culture negative**	78	5.1	31	3.4	109	4.5
***M. tuberculosis* culture positive**	1422	93.3	847	94.0	2269	93.6
**Drug sensitivity test (DST) details**						
**DST not done**	202	14.2	133	15.7	335	14.8
**DST available**	1220	85.8	714	84.3	1934	85.2

A six-month financial interruption and loss of staff necessitated freezing cultures; DST non-availability was caused by failure to subsequently recover *M. tuberculosis* from the frozen cultures. Comparison of the distribution of patients with unrecoverable cultures and no DST results showed no significant differences by sex, age, NSP or PT and HIV status (S1 Table).

Among NSP cases, 976 (80%) had *M. tuberculosis* susceptible to all four drugs tested, while 244 (20%) showed any resistance to the drugs tested. Among patients with any resistance, 10.4% each were resistant to INH and OFX. The prevalence of any RMP resistance was 2.6% while MDR-TB was seen in 1.8%. The proportion of mono resistance to OFX (8.8%) and to INH (7.5%) was high compared to RMP (0.7%). Pre XDR-TB (MDR-TB isolates resistant to either KAN or OFX) was 0.4%. None exhibited mono resistance to KAN (Table 2).

**Table 2:** The prevalence of resistance to first and second line anti-TB drugs among *M*. *tuberculosis* isolates from new and previously treated cases of tuberculosis.

	**New cases**		**Previously treated Cases**	
	**(n = 1220)**		**(n = 714)**	
	**Numbers (%)**	**95% CI**	**Numbers (%)**	**95% CI**
**Susceptible to all drugs**	976 (80.0)	77.8–82.2	444 (62.2)	58.5–65.7
**Any Resistance**				
Isoniazid	127 (10.4)	8.7–12.1	214 (30.0)	26.7–33.4
**Rifampicin**	**32 (2.6)**	**1.7–3.5**	**108 (15.1)**	**12.6–17.9**
Kanamycin	1 (0.1)	0.0–0.2	7 (1.0)	0.3–1.9
Ofloxacin	127 (10.4)	8.7–12.1	99 (13.9)	11.4–16.5
**Mono Resistance**				
Isoniazid	91 (7.5)	6.0–8.9	95 (13.3)	10.8–15.8
Rifampicin	8 (0.7)	0.2–1.1	11 (1.5)	0.6–2.4
Kanamycin	0.0	0.0	1 (0.1)	0.0–0.4
Ofloxacin	107 (8.8)	7.2–10.4	41 (5.7)	4.0–7.4
**Combined resistance**				
Ofloxacin +Rifampicin	2 (0.2)	0.0–0.4	3 (0.4)	0.0–0.9
Ofloxacin + Isoniazid	14 (1.1)	0.5–1.7	24 (3.4)	2.0–4.7
Isoniazid+Kanamycin	0	0.0	1 (0.1)	0.0–0.4
**MDR**	**22 (1.8)**	1.1–2.5	**94 (13.2)**	10.7–15.6
Isoniazid +Rifampicin	17 (1.4)	0.7–2.1	62 (8.7)	6.6–10.7
Isoniazid + Rifampicin + Ofloxacin (Pre-XDR)	4 (0.3)	0.0–0.6	27 (3.8)	2.4–5.2
Isoniazid + Rifampicin + Kanamycin (Pre-XDR)	1 (0.1)	0.0–0.2	1 (0.1)	0.0–0.4
Isoniazid +Rifampicin + Ofloxacin +Kanamycin **(XDR-TB)**	0	-	**4 (0.6)**	**0.0–1.1**

Among PT cases, 444 (62.2%) had drug sensitive TB and 270 (37.8%) had resistance to at least to one of the four drugs tested. In this group, any INH resistance was 30%. Mono resistance was 13.3% to INH, 5.7% to OFX and 1.5% to RMP. The prevalence of any RMP resistance was 15.1% while MDR-TB was 13.2%. The prevalence of pre-XDR in PT cases was 3.9%. As a proportion of MDR-TB, however, additional quinolone resistance was observed in 4/22 NSP and 27/94 PT patients respectively. Four PT patients had XDR-TB (0.6%) (Table 2).

Among previously treated patients, the prevalence of MDR-TB among treatment failure (A patient whose sputum smear or culture is positive at 5 months or later during treatment), treatment after default (A patient whose treatment was interrupted for 2 consecutive months or more) and relapse (A patient who was declared cured or treatment completed, but who reports back to the health service and is now found to be sputum smear positive) groups were 35%, 10% and 13%, respectively. The prevalence of MDR-TB among the treatment failure group was significantly higher than the treatment after default (Odds ratio 4.62, 95% CI 2.38–8.96) and relapse (Odds ratio 3.49, 95% CI 1.82–6.69) groups.

Mono and any INH resistance were frequently seen among all types (treatment failures [16.4% and 54.5%, respectively], treatment after default [11.5% and 25.2%, respectively] and relapse [15.6% and 31.9%, respectively]) of PT patients. In the PT group, any RMP resistance was significantly higher in females than in males (21% vs. 13.9%; p value 0.05). Any RMP resistance was also higher among PT group in older (>45 years) than in younger age group (18.1% vs. 13.3%; p value 0.01) with a. However, such differences were not seen in NSP cases.

The HIV status of smear positive patients was available for 2232 (92%) of 2425 patients enrolled in the study. A total of 112 out of 2232 (5%) cases were found to be HIV positive (95% CI 4.1–5.9). HIV positivity was 5% each for NSP (72 out of 1436; 95% CI 3.9–6.1) and PT cases (40 out of 796; 95% CI 3.5–6.5). The prevalence of MDR-TB was 2.7% and 5% respectively, among HIV positive and HIV negative patients (S2 Table). None of the HIV positive patients had XDR-TB.

## Discussion

The present survey is the second largest population-based drug resistance survey in India, reporting resistance levels to four key anti-TB drugs, among TB patients enrolled and treated under the RNTCP in Tamil Nadu. No nationally representative drug resistance survey has been undertaken in India, though one is planned for 2014. Between 2007 and 2009, population based, representative drug resistance surveillance studies in three states of India (Gujarat, Maharashtra and Andhra Pradesh) were conducted by the Central TB Division following the standard international protocol. These studies revealed that the burden of MDR-TB among NSP cases was between 1.8% and 2.7% [13]. A previous survey in Tamil Nadu in 1997 had shown that the prevalence of any RMP resistance was 4.4% among NSP cases [8]. However, that study was done using a different protocol and is therefore not strictly comparable.

Our survey found a prevalence of 1.8% MDR-TB among NSP which is lower than what was reported for Gujarat in 2007 (2.4%) [7]. The prevalence is lower than that reported from other high TB burden countries such as the former Soviet Union (ranged from 2.8% to 28.6%) [14], China (ranged from 3.8% to 14.7%) [15, 16], and some low burden countries: Cameroon (6.67%) [17], Turkey (6.6%) [6] and Kenya (6.8%) [18]. All these surveys were done between 2008 and 2012 and appropriate guidelines were followed.

Among NSP patients, the prevalence of any INH resistance (10.4%), any RMP resistance (2.6%) and MDR-TB (1.8%) was similar to levels reported earlier between 1992 and 2004 from the southern parts of India [8,19–21]. The levels of mono resistance to INH (7.5%) and RMP (0.7%) are lower than the available reports from different regions of India [22,23]. The level of OFX mono resistance was 8.8% among NSP cases.

Cartridge based nucleic acid amplication test (CB NAAT) is being scaled up in India to test patients suspected to have drug resistance. The detection of RMP resistance is considered as a surrogate marker for MDR-TB [24] and such patients are treated for MDR-TB. With the inclusion of cases with mono resistance to RIF, the prevalence of RMP resistance among NSP and PT cases increased to 2.6% and 15.1% respectively.

The prevalence of MDR-TB among PT cases was lower (13.2%) than in a previous survey done in the state of Gujarat in 2007 (17.4%). However, any INH resistance (30%) and any OFX resistance (13.9%) were high among PT cases. Any OFX resistance was also observed in 10.4% of NSP cases. This could be attributed to the common use of flouroquinolones for the treatment of respiratory and other infections in the community. Studies from other countries have shown that OFX resistance varies from 0.6 to 1.8% among NSP and 4.1 to 9.3% among PT cases [25–27]. Likewise a study from South-West Nigeria in 2011 had shown that OFX resistance among NSP and PT cases was 4.3% and 9.1% respectively [28]. A worrying finding was that 18.2% of new and 28.7% of PT patients with MDR-TB had additional OFX resistance, and in view of cross-resistance between the quinolones, suggest the need to review levofloxacin-containing regimens currently being used to treat MDR-TB.

The earlier drug resistance survey in Gujarat showed a prevalence of 4% of XDR-TB among MDR-TB isolates [7]. The present study also showed a prevalence of 4.3% of XDR-TB among known MDR-TB isolates. In both of these studies, no XDR-TB was detected among new TB patients.

In the present study, among PT cases, females had a significantly higher rate of MDR-TB than males. Studies from South Africa, where TB and HIV were endemic [29] and Estonia [30] also reported a greater proportion of MDR-TB in women than in men. High rates of MDR-TB among women could be related to high rates of treatment failures; however, the reasons for this phenomenon need further exploration. Similarly, high rates of MDR-TB among older patients could be related to their previous repeated chemotherapy and accompanying co-morbidities, which need further exploration. The fact that MDR-TB rates were no different between HIV positive and negative patients confirms previous reports that HIV is not a risk factor for drug resistance in India [31].

The inability to perform DST for 14.8% (14.2% NSP and 15.7% PT cases) of cultures was due to interruption in receiving financial support, non-availability of laboratory technicians to undertake DST for a period of 6 months and failure to subsequently recover *M. tuberculosis* from the frozen cultures. While very concerning, scrutiny of the distribution of clinical and demographic characteristics of these cases, including prior treatment status, suggested that selection bias due to this operational gap was unlikely.

In this study, for the first time, direct sputum specimens were transported without CPC preservative and cold chain management, and processed for culture in the laboratory. Transportation challenges were successfully mitigated by the supply of sterile pre-packed plastic containers for the collection and transportation of sputum specimens at the beginning of the study. The observed high recovery rate of *M. tuberculosis* from the specimens (93.6%) suggests procedures were adequate and highly encouraging.

Collection of three samples (spot—morning—spot) or two samples (spot—morning) necessitates multiple visits by the patients, which leads to high drop out of infectious cases [32]. Keeping this in mind, two spot sputum specimens were collected within 2–3 hours from smear positive PTB patients in the health facilities. Moreover, the recovery of *M. tuberculosis* from study patients using both spot specimens or either specimen showed no difference (McNemar’s test value is 1.114). This suggests that for DR surveys, one additional spot specimen is sufficient from smear positive patients to achieve valid results, a strategy that would simplify logistics. However, this is feasible only if all precautions and strict monitoring activities are ensured by the DRS team for proper collection and transportation of sputum samples.

RNTCP is the largest TB control program globally, treating over 1.5 million patients each year. Periodic surveys provide useful information on rates of drug resistance to various anti-TB drugs in different patient categories as well as in monitoring the performance of the program. Our findings provide reassurance that MDR-TB rates have remained stable in this region, but highlight the high rates of OFX resistance and ‘pre-XDR-TB’, due most likely to the widespread misuse of fluoroquinolones.

## Conclusions

The survey provides data on first and second line drug resistance among a representative group of patients treated under programmatic conditions. Considering RMP mono resistance as a surrogate marker for MDR-TB, the level of any RMP resistance among NSP and PT cases were 2.6% and 15.1% respectively. Additional OFX resistance was seen among 18.2% of new and 28.7% of PT MDR-TB patients. HIV status of the patients had no impact on drug resistance levels. While overall drug resistance rates appear stable, action is required to minimise the misuse of fluoroquinolone for the treatment of other common infections, as it might impact their effectiveness in regimens for the treatment of drug sensitive and drug resistant TB.

## Supporting Information

S1 Table 1A—The age and sex wise distribution of DST has not done Vs DST available among new and previously treated cases of tuberculosis. **1B**—The DMC wise comparison of DST has not done Vs DST available among new and previously treated cases of tuberculosis.
(DOC)

S2 Table Age and sex wise comparison of MDR-TB cases among HIV positive and negative cases. (DOC)

## References

[1] Raviglione M, Marais B, Floyd K, Lonnroth K, Getahun H, et al (2012) Scaling up interventions to achieve global tuberculosis control: progress and new developments. Lancet 379: 1902–1913. 10.1016/S0140-6736(12)60727-2 22608339

[2] WHO (2005) Global tuberculosis control: surveillance, planning, financing. WHO/HTM/TB/2005.349. World Health Organization, Geneva.

[3] WHO (2009) Global tuberculosis control: epidemiology, strategy, financing. WHO/HTM/TB/2009.411. World Health Organization, Geneva.

[4] WHO (2012) Global tuberculosis report. WHO/HTM/TB/2012.6. World Health Organization, Geneva.

[5] WHO (2010) Multidrug and extensively drug-resistant TB (M/XDR-TB): 2010 global repoPT on surveillance and response. WHO/HTM/TB/2010.3. World Health Organization, Geneva.

[6] Kart L, Altin R, Tor M, Gulmez I, Oymak SF, et al (2002) Antituberculosis drug resistance patterns in two regions of Turkey: a retrospective analysis. Ann Clin Microbiol Antimicrob 1: 6 12537590 10.1186/1476-0711-1-6 PMC149381

[7] Ramachandran R, Nalini S, Chandrasekar V, Dave PV, Sanghvi AS, et al (2009) Surveillance of drug-resistant tuberculosis in the state of Gujarat, India. Int J Tuberc Lung Dis 13: 1154–1160. 19723407

[8] Paramasivan CN, Bhaskaran K, Venkataraman P, Chandrasekaran V, Narayanan PR (2000) Surveillance of drug resistance in tuberculosis in the state of Tamil Nadu. Indian J Tuber 47: 27–33.

[9] WHO (2009b) Guidelines for surveillance of drug resistance in tuberculosis. Fourth Edition. WHO/HTM/TB/2009.422. World Health Organization, Geneva.

[10] CTD (2005) Central TB Division: Module for laboratory technician. Second edition, October, 2005. Available: http://www.tbcindia.nic.in/pdfs/Module%20for%20Laboratory%20Technician.pdf. Accessed 2013 Aug 20.

[11] Central TB Division (CTD 2009). Revised National TB Control Programme Training Manual for Mycobacterium tuberculosis Culture & Drug susceptibility testing. Available: http://tbcindia.nic.in/pdfs/Training%20manual%20M%20tuberculosis%20C%20DST.pdf. Accessed 2014 Nov 24.

[12] WHO (2001) Guidelines for drug susceptibility testing for second-line anti-tuberculosis drugs for dots-plus. WHO/CDS/TB/2001.288. World Health Organization, Geneva.

[13] CTD (2011) Central TB Division: National scale-up plan for the programmatic management of drug-resistant tuberculosis (PMDT). March, 2011. Available: http://www.tbcindia.nic.in/pdfs/National%20PMDT%20Scale-up%20Plan%20-%20India%20-%202011-12.pdf. Accessed 2013 Aug 20.

[14] WHO (2008) Anti-tuberculosis drug resistance in the world. FouPTh global repoPT. WHO/HTM/TB/2008.394. World Health Organization, Geneva.

[15] Hu Y, Mathema B, Wang W, Hoffner S, Kreiswirth B, et al (2008) Prevalence of multidrug-resistant pulmonary tuberculosis in counties with different duration of DOTS implementation in rural China. Microb Drug Resist 14: 227–232. 10.1089/mdr.2008.0823 18707239

[16] Zhao Y, Xu S, Wang L, Chin P, Wang S, et al (2012). National Survey of Drug-Resistant Tuberculosis in China. N Engl J Med 366: 2161–2170. 10.1056/NEJMoa1108789 22670902

[17] Assam-Assam JP, Penlap VB, Cho-Ngwa F, Tedom JC, Ane-Anyangwe I, et al (2011) Mycobacterium tuberculosis complex drug resistance pattern and identification of species causing tuberculosis in the West and Centre regions of Cameroon. BMC Infect Dis 11: 94 10.1186/1471-2334-11-94 21496268 PMC3090333

[18] Klinkenberg E, van den Hof S, Tursynbayeva A, Kipruto H, Wahogo J, et al (2012) Integration of HIV testing in tuberculosis drug resistance surveillance in Kazakhstan and Kenya . Int J Tuberc Lung Dis 16: 615–617. 10.5588/ijtld.11.0262 22409816

[19] Chandrasekaran S, Jagota P, Chaudhuri K (1992) Initial drug resistance to anti-tuberculosis drugs in urban and rural district tuberculosis programme. Indian J Tuber 39: 171–175.

[20] Paramasivan CN, Venkataraman P, Chandrasekaran V, Bhat S, Narayanan PR (2002) Surveillance of drug resistance in tuberculosis in two districts of South India. Int J Tuberc Lung Dis 6: 479–484. 12068979 10.5588/09640569512977

[21] Paramasivan CN, Venkataraman P (2004) Drug resistance in tuberculosis in India. Indian J Med Res 120: 377–386. 15520487

[22] Gupta PR, Singhal B, Sharma TN, Gupta RB (1993) Prevalence of initial drug resistance in tuberculosis patients attending a chest hospital. Indian J Med Res 97: 102–103. 8406629

[23] Mahadev B, Kumar P, Agarwal SP, Chauhan LS, Srikantaramu N (2005) Surveillance of drug resistance to anti-tuberculosis drugs in districts of Hoogli in West Bengal and Mayurbhanj. Indian J Tuber 52: 5–10.

[24] Panayotis I, Dimitrios P, Simona K, Stavroula N, Marina P, et al (2011) Cepheid Gene Xpert MTB/RIF Assay for *Mycobacterium tuberculosis* Detection and Rifampin Resistance Identification in Patients with Substantial Clinical Indications of Tuberculosis and Smear-Negative Microscopy Results. J Clin Micro 49: 3068–3070. 10.1128/JCM.00718-11 21677069 PMC3147726

[25] Ginsburg AS, Woolwine SC, Hooper N, Benjamin WH Jr, Bishai WR, et al (2003) The rapid development of fluoroquinolone resistance in M. tuberculosis. N Engl J Med 349: 1977–1978. 14614180 10.1056/NEJM200311133492023

[26] Umubyeyi AN, Rigouts L, Shamputa IC, Fissette K, Elkrim Y, et al (2007) Limited fluoroquinolone resistance among Mycobacterium tuberculosis isolates from Rwanda: results of a national survey. J Antimicrob Chemother 59: 1031–1033. 17329272 10.1093/jac/dkm038

[27] Bozeman L, Burman W, Metchock B, Welch L, Weiner M (2005) Flouroquinolones susceptibility among Mycobacterium tuberculosis from the United States and Canada. Clin Infect Dis 40: 386–391. 15668861 10.1086/427292

[28] Daniel O, Osman E, Bakare R, Adebiyi P, Ige O, et al. (2011) Ofloxacin resistance among Mycobacterium tuberculosisisolates in two states of south-west Nigeria.African J Res Med 2011 March: 18–20.

[29] O'Donnell MR, Zelnick J, Werner L, Master I, Loveday M, et al (2011).Extensively drug-resistant tuberculosis in women, Kwazulu-natal, South Africa. Emer Inf Dis 17: 1942–1945. 10.3201/eid1710.110105 PMC3310667 22000378

[30] Blöndal K, Rahu K, Altraja A, Viiklepp P, Rahu M (2013) Overall and cause-specific mortality among patients with tuberculosis and multidrug-resistant tuberculosis. Int J Tuberc Lung Dis 17: 961–968. 10.5588/ijtld.12.0946 23743316

[31] Swaminathan S, Paramasivan CN, Ponnuraja C, Iliayas S, Rajasekaran S, et al (2005) Anti-tuberculosis drug resistance in patients with HIV and tuberculosis in South India . Int J Tuberc Lung Dis 9: 896–900. 16104637

[32] Myneedu VP, Verma AK, Sharma PP, Behera D A (2011) A pilot study of same day sputum smear examination, its feasibility and usefulness in diagnosis of pulmonary TB. Indian J Tuberc 58: 160–167. 22533165

