# Supplementary material for: High Rates of Ofloxacin Resistance in Mycobacterium tuberculosis among Both New and Previously Treated Patients in Tamil Nadu, South India
Source: PLoS One. 2015 Mar 4;10(3):e0117421. doi: 10.1371/journal.pone.0117421 (PMC4349813; doi:10.1371/journal.pone.0117421)
Supplement: S1 Table — 1B—The DMC wise comparison of DST has not done Vs DST available among new and previously treated cases of tuberculosis. (DOC) [file pone.0117421.s001.doc]

**Supplementary table 1**

**1A - The age and sex wise distribution of DST has not done Vs DST available among new and previously treated cases of tuberculosis.**

|  | | **New cases** | | **Previously**  **treated cases** | |
| --- | --- | --- | --- | --- | --- |
|  |  | **DST not done** | **DST available** | **DST not done** | **DST available** |
| **Age** | ≤ 45 Years | 111 | 657 | 71 | 421 |
|  | ≥ 46 Years | 91 | 541 | 61 | 288 |
| **Total** | | **202** | **1198*** | **132*** | **709*** |
|  | | **P value 0.98** | | **P value 0.23** | |
| **Sex** | Female | 49 | 274 | 17 | 124 |
|  | Male | 153 | 946 | 116 | 590 |
| **Total** | | **202** | **1220** | **133** | **714** |
|  | | **P value 0.57** | | **P value 0.19** | |
| * The detail of age is not available for few patients. Hence the data cannot be matched with data presented in Table 1 and 2. | | | | | |

**1B – The DMC wise comparison of DST has not done Vs DST available among new and previously treated cases of tuberculosis.**

| **New cases** | | | | **Previously treated cases** | | | |
| --- | --- | --- | --- | --- | --- | --- | --- |
| **No. of DMCs** | **No. of cases missed** | **DST** | | **No. of DMCs** | **No. of cases missed** | **DST** | |
|  |  | **Not done** | **Done** |  |  | **Not done** | **Done** |
| 8 | 0 | 0 | 73 | 20 | 0 | 0 | 62 |
| 12 | 1 | 12 | 228 | 17 | 1 | 17 | 129 |
| 14 | 2 | 28 | 257 | 8 | 2 | 16 | 54 |
| 12 | 3 | 36 | 234 | 6 | 3 | 18 | 111 |
| 8 | 4 | 32 | 168 | 3 | 4 | 12 | 77 |
| 5 | 5 | 25 | 84 | 2 | 5 | 10 | 39 |
| 4 | 6 | 24 | 67 | 1 | 7 | 7 | 20 |
| 1 | 7 | 7 | 14 | 1 | 9 | 9 | 41 |
| 2 | 8 | 16 | 48 | 1 | 12 | 12 | 76 |
| 1 | 9 | 9 | 14 | 1 | 15 | 15 | 50 |
| 1 | 13 | 13 | 33 | 1 | 17 | 17 | 55 |
| **68** |  | **202** | **1220** | **61*** |  | **133** | **714** |
| Chi square test was performed for more than 5 number of cases missed and showed a value of 3.24 (p value <0.66) | | | | Chi square test was performed for more than 5 number of cases missed and showed a value of 3.93 (p value <0.55) | | | |
| ***** 7 DMCs were not assigned for recruitment of previously treated cases of tuberculosis. | | | | | | | |
