# Supplementary material for: High Rates of Ofloxacin Resistance in Mycobacterium tuberculosis among Both New and Previously Treated Patients in Tamil Nadu, South India
Source: PLoS One. 2015 Mar 4;10(3):e0117421. doi: 10.1371/journal.pone.0117421 (PMC4349813; doi:10.1371/journal.pone.0117421)
Supplement: S2 Table — (DOC) [file pone.0117421.s002.doc]

**Supplementary table 2: Age and sex wise comparison of MDR-TB cases among HIV positive and negative cases.**

|  |  | **HIV Positive**  **(No. of MDR)** | **HIV Negative**  **(No. of MDR)** |
| --- | --- | --- | --- |
| **Age** | ≤ 45 Years | 91 (2) | 1145 (56) |
|  | ≥ 46 Years | 19 (1) | 949 (51) |
| Total | | 110* (3) | 2094^#^ (107) |
| **Sex** | Female | 30 (0) | 446 (27) |
|  | Male | 82 (3) | 1674 (80) |
| Total | | 112 (3) | 2120 (107) |
| * The detail of age is not available for 2 patients; # The detail of age is not available for 26 patients. | | | |
